# Construction of Gene Modules and Analysis of Prognostic Biomarkers for Cervical Cancer by Weighted Gene Co-Expression Network Analysis

**DOI:** 10.3389/fonc.2021.542063

**Published:** 2021-03-18

**Authors:** Jiamei Liu, Shengye Liu, Xianghong Yang

**Affiliations:** ^1^Department of Pathology, The Shengjing Hospital of China Medical University, Shenyang, China; ^2^Department of Orthopedics, The Shengjing Hospital of China Medical University, Shenyang, China

**Keywords:** cervical cancer, weighted gene co-expression network analysis (WGCNA), The Cancer Genome Atlas (TCGA), modules, hub genes

## Abstract

**Background:**

Despite advances in the understanding of neoplasm, patients with cervical cancer still have a poor prognosis. Identifying prognostic markers of cervical cancer may enable early detection of recurrence and more effective treatment.

**Methods:**

Gene expression profiling data were acquired from the Gene Expression Omnibus database. After data normalization, genes with large variation were screened out. Next, we built co-expression modules by using weighted gene co-expression network analysis to investigate the relationship between the modules and clinical traits related to cervical cancer progression. Functional enrichment analysis was also applied on these co-expressed genes. We integrated the genes into a human protein-protein interaction (PPI) network to expand seed genes and build a co-expression network. For further analysis of the dataset, the Cancer Genome Atlas (TCGA) database was used to identify seed genes and their correlation to cervical cancer prognosis. Verification was further conducted by qPCR and the Human Protein Atlas (HPA) database to measure the expression of hub genes.

**Results:**

Using WGCNA, we identified 25 co-expression modules from 10,016 genes in 128 human cervical cancer samples. After functional enrichment analysis, the magenta, brown, and darkred modules were selected as the three most correlated modules for cancer progression. Additionally, seed genes in the three modules were combined with a PPI network to identify 31 tumor-specific genes. Hierarchical clustering and Gepia results indicated that the expression quantity of hub genes NDC80, TIPIN, MCM3, MCM6, POLA1, and PRC1 may determine the prognosis of cervical cancer. Finally, TIPIN and POLA1 were further filtered by a LASSO model. In addition, their expression was identified by immunohistochemistry in HPA database as well as a biological experiment.

**Conclusion:**

Our research provides a co-expression network of gene modules and identifies TIPIN and POLA1 as stable potential prognostic biomarkers for cervical cancer.

## Introduction

Cervical cancer (CC) is of lethal malignancy in the female population. Although prognosis can be improved by early detection, cervical cancer still ranks second in morbidity and becomes the leading cause of death in women. It accounts for 10%–15% of tumor-related deaths in women worldwide, with about 527,600 new cases and over 250,000 deaths per year (1,2). Although more than 90% of patients with early-stage CC can be cured, the prognosis of patients with advanced CC, especially patients with CC metastasis, is quite poor (3,4). Therefore, early and accurate diagnosis of cervical cancer and discovery of new therapeutic targets would greatly improve the survival rate and prognosis of cervical cancer patients. Numerous studies have examined the relationship between genes and proteins in CC. Discovery of early stage cervical cancer biomarkers will reveal new information regarding the pathogenesis of CC; modulation of tumor proliferation, invasion, and metastasis; guide clinical treatment; and allow for a more accurate determination of prognosis.

Recent advancements in advanced gene interaction network approaches have provided researchers with opportunities to explore possible intrinsic links among functional gene clusters. Weighted gene co-expression network analysis (WGCNA) is used to rebuild robust gene co-expression networks, referred to as modules according to large-scale gene expression profiles. They enable to distinguish hub genes that are centrally located and drive key cellular signaling pathways (5,6). The WGCNA methodology has become functional interpretation instruments in systems biology and has initiated new fields in the pathophysiology of cancer. Identification of meaningful modules related to cancer grades and stages will allow greater understanding of tumor mechanisms and prognosis, as well as promote discovery of novel diagnostic and therapeutic direction. This research is exactly based on the theoretical basis above.

**Figure 1:**
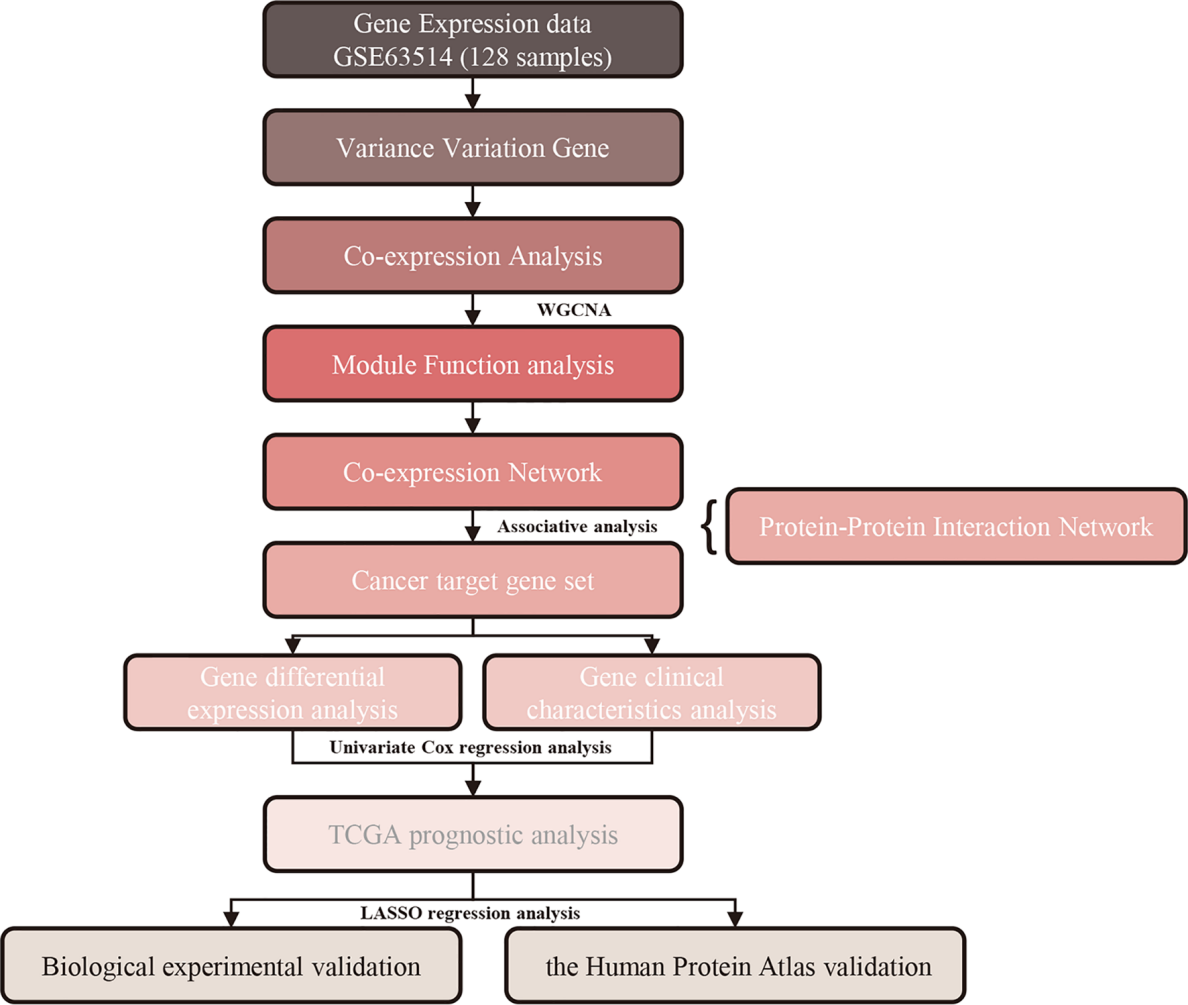
Study workflow. WGCNA, weighted gene co‐expression network analysis.

In this study, we conducted a WGCNA and calculated module-trait correlations based on the GEO and TCGA databases, which contain 128 cervical cancer samples. Through this approach, we identified meaningful co-expression modules significantly related to tumor grade and stage. We also discovered hub genes through KEGG enrichment analysis, hierarchical clustering analysis, and least absolute shrinkage and selection operator (LASSO) regression analysis. These hub genes may serve as potential diagnostic and prognostic biomarkers of cervical cancer and may potentially be targeted by novel therapies. Furthermore, the expression levels of hub genes were validated by quantitative polymerase chain reaction (qPCR) in cervical cancer cell lines and immunohistochemistry in the Human Protein Atlas online database. The workflow of this study is displayed in **Figure 1**.

## Materials and Methods

### Data Source and Screening of Genes

The expression profiling data were derived from 128 cervical cancer samples of different stages and related clinical information [GEO: GSE63514] (7). The data were pretreated with normalized GC-RMA values and base 2 log transformed. First, the probes corresponding to the genes with idle probes were removed. The median was calculated as the expression level of the gene when multiple probes are homologous to one same gene. Then, we figured out the coefficient of variation (CV) of each gene in different samples and chose the first 50% of the genes with the largest CV as the target genes for subsequent analysis.

### Construction of WGCNA and Co-Expression Module Detection

First, we calculated the absolute value of the Pearson correlation coefficient between two genes to construct the similarity matrix of their individual gene expression quantity. Next, we converted the gene expression matrix into an adjacency matrix with a signed network type and exponent β. The definition of the adjacency matrix A_ij_ is as follows: , S_ij_ = Pearson’s correlation between gene i and gene j, A_ij_=contiguity of gene i and gene j. Taking the Pearson correlation coefficient at the exponential level of each pair of genes would strengthen the strong correlation and weaken the inferior correlation. Next, the adjacency matrix was transformed into a topological matrix. The degree of association between genes is described by a topological overlap matrix (TOM). Hierarchical clustering analysis was carried out for further module identification using the dynamic shear method. It shows the iterative grouping or splitting of clusters in a hierarchical tree. A dendrogram can offer preliminary judgment and provide a visualization of the distances between subclusters (8). Gene significance (GS) is used to measure the connective degree of genes and their external information; higher GS indicates more biological significance. If GS=0, the gene is not involved in the biological process.

We used WGCNA as a systematic biological approach to build a scale-free network to examine potential gene correlations within the gene expression dataset. Based on the target genes obtained in the previous step, we used the WGCNA function (https://www.r-project.org/) in R to construct a WGCNA and identify co-expression modules (8). In the co-expression network, the module eigengene (ME) is defined as the first principal component (PC) of each module’s gene expression matrix, which represents the highest percent of variance for the expression values of all module genes in a sample (9,10). The signed weighted network analysis was adapted to the cervical cancer dataset.

### Functional Enrichment Analysis of Co-Expression Modules

In order to explore the gene-associated functions of co-expression modules, we used the clusterProfiler function in R (11) and subjected the modules to the Kyoto Encyclopedia of Genes and Genomes pathway analysis (KEGG).

### Correlation Analysis Between the Modules and Cancer Samples

According to the characteristics of the samples in GSE63514, one hundred and twenty-eight samples were divided into four stages (low grade, moderate grade, high grade, and epithelial cancer). Afterwards, we define the corresponding-attribute sample as 1; other samples are defined as 0. Then, normal samples were collaborated with four stages of cancer-related samples to form a cancer progression gradient and construct a phenotypic vector. A phenotypic matrix was built up based on the process above. Eventually, we calculated the correlation between each module and various factors in the phenotypic matrix, and screened out cancer-related modules.

### Construction of WGCNA in Cancer-Related Modules

According to the relationships between the expressed genes in the co-expression modules, the co-expression network of cancer-related modules was derived with an express weight value greater than 0.1, which was regarded as the final total express network edge. Furthermore, we downloaded all the protein-protein interaction (PPI) network data in HIPPIE v2.0 (12) to expand the network, mapped cancer-related modules to the human PPI network, and finally obtained the co-expression and interaction specific networks.

### Expression Profiling and Functional Enrichment Analysis of Specific Genes

In order to observe the relationships between specific genes, we analyzed the expression profiling data by expression profiling hierarchical clustering analysis and utilized KEGG Pathway enrichment analysis for further functional enrichment analysis.

### Relationships Between Cancer-Specific Genes and Clinical Prognosis

To further analyze gene expression changes and prognosis related to high-abundance genes in cervical cancer, we downloaded the RNA-Seq datasets with clinical follow-up information, a total of 304 samples of cervical cancer, and convert FPKM to TPM. After being normalized by GAPDH and z-score conversions respectively, univariate Cox regression analysis for each candidate gene was used for relationship of prognosis. Gepia (http://gepia.cancer-pku.cn/) (13), an online tool, was applied to mine the change in expression of cancer specific genes and prognosis using data from The Cancer Genome Atlas. In order to maintain high accuracy, we used the glmnet function in R to construct a prognostic signature by LASSO regression analysis.

### Experimental Validation of Two Prognostic Genes

Human cervical cancer cell lines, SIHA and HELA, were maintained in a DMEM high-glucose medium. Human cervical cancer intestinal-metastatic cell line CASKI were cultured in an RPMI-1640 medium. The Medium were all supplemented with a 10% FBS, 100 µg/ml streptomycin, and 100 U/ml penicillin at 37˚C with 5% CO2 in a humidified atmosphere. Quantification of gene expression measurement is carried out by quantitative polymerase chain reaction as previously cited. The primer sequences were as follows: forward, 5’-AGAACCACAGGAGAATGGCG-3’ and reverse, 5’-GTACACGAACAGGTGCTCCA-3’ for TIPIN; forward, 5’-CCTCACTGCAAGTCAGAGGG-3’ and reverse, 5’-ATCTGCTGGGCCAGGTAGTA-3’ for POLA1; forward, 5’-TCAAGGCTGAGAACGGGAAG-3’ and reverse, 5’-TGGACTCCACGACGTACTCA-3’ for GAPDH.

### Immunohistochemistry Validation of Hub Gene

The HPA database was further used to illustrate the distribution and subcellular localization of relative proteins in CC tissues. Altered gene expression patterns can also be validated at the protein level. Clinical specimens of cervical cancer from HPA were available and convenient for further confirmation of aforementioned results; hub gene protein expression could be analyzed with an optic microscope.

## Results

### Data Preprocessing and Sample Classification

Gene expression profiling data from cervical cancer patients were downloaded from GEO in the National Center for Biotechnology Information database (http://www.ncbi.nlm.nih.gov/geo/). We used the mRNA from the internal testing dataset GSE63514 with the full human genome Affymetrix Human Genome U133 Plus 2.0 Array. The dataset contained 128 cervical specimens, separated into four disease stages according to histopathology: normal (24 samples), CIN1 (14 samples), CIN2 (22 samples), CIN3 (40 samples), and tumors (28 samples). Patient clinical information and survival time were also included.

The coefficient of variation (CV) for each probe was calculated and screened for all samples. 10,939 genes were obtained from 21,879 genes in total (**Supplementary Table 1**). Genes with a CV in top 20 were selected as the seed genes.

### Modules in Gene Co-Expression Network Functional Enrichment Analysis

**Figure 2:**
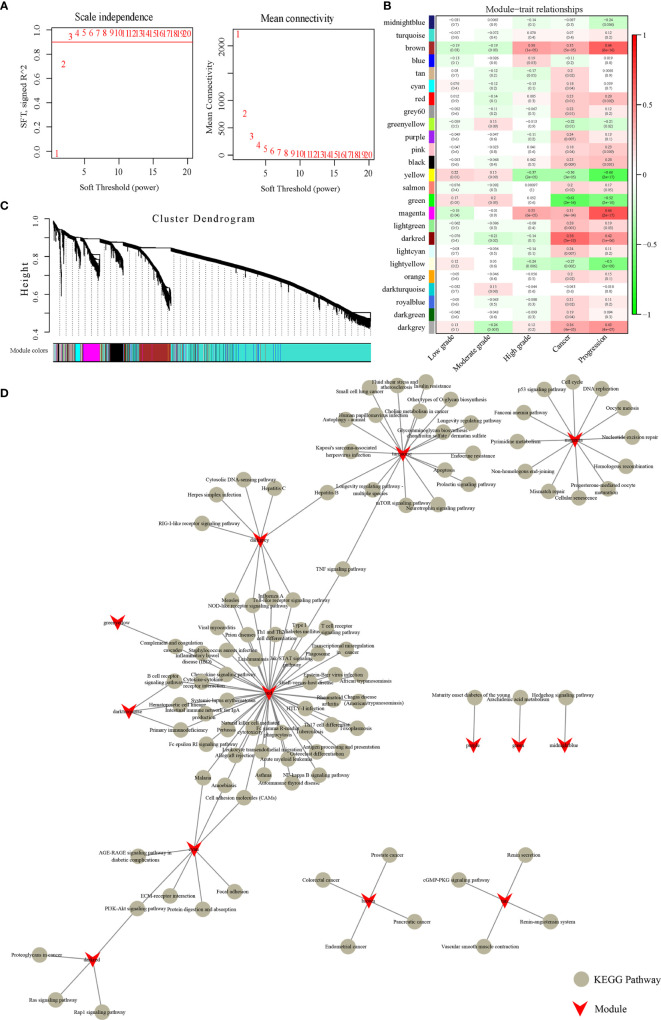
Weighted gene co-expression network analysis. **(A)** Analysis of network topology for various soft-thresholding powers. **(B)** Module-trait relationships. Each row corresponds to a module eigengene, and each column corresponds to a trait. Each cell contains the corresponding correlation coefficient and *p*-value. The cells are color-coded according to the degree of correlation, and the correlation coefficients decrease from red to green. **(C)** Gene dendrogram and module colors. Clustering dendrograms of genes, with dissimilarity based on topological overlap, are bracketed together with assigned module colors. Twenty-five co-expression modules were established and illustrated in different colors. **(D)** KEGG Pathway analysis of 13 modules.

We used a network built by a scale-free WGCNA to examine the potential gene correlation structures within the gene expression data. Seed genes with obvious variation and prognostic significance were used for network building based on the TOM function in R. We then clustered the genes using the average-linkage hierarchical clustering method. In accordance with the standards of hybrid dynamic shearing, the number of each gene network module was set to at least 30. The results showed that the co-expression network was scale-free. To further ensure that the network was a scale-free type, we implemented β=3 as the soft-thresholding parameter (**Figure 2A**). Highly connective module genes are represented and summarized by ME. The expression matrix was converted into an adjacency matrix, and then further converted into a topology matrix. Modules were subjected to clustering analysis, where modules at close range will be merged into a new module. A total of 25 modules with 25 different colors are shown in **Figure 2B**, and the 10,016 genes allocated into the modules are listed in **Supplementary Table 2**. The gray module is a collection of genes that cannot be clustered into other modules. As shown in **Figure 2C**, there is a low correlation between the early stage samples and the various modules. The magenta and brown modules had the greatest association with both cervical cancer and stage, and the darkred module had the highest relevancy to the cancer stage. Overall, the magenta, brown, and darkred modules are the most relevant modules correlated with cervical cancer development.

Next, we used the clusterProfiler function in R to conduct KEGG enrichment analysis on the genes in each module, and the results are listed in **Supplementary Table 3**. There were 13 modules enriched to 114 KEGG Pathways, but there were few common pathways among the different modules (**Figure 2D**). This suggests that these modules are independently functional. Furthermore, it was shown that the brown module was connected with cancer and progression, and was enriched in four cancer pathways: endometrial cancer, colorectal cancer, prostate cancer, and pancreatic cancer. The darkred module was enriched in four pathways: the PI3K-Akt signaling pathway, Rap1 signaling pathway, proteoglycans in cancer, and Ras signaling pathway, which are closely associated with cancer progression. Magenta enriched to 11 KEGG pathways, including cell cycle, DNA replication, and the p53 signaling pathway, among others. These results suggest that the magenta, brown, darkred modules are likely to be closely related to cancer progression.

### Establishment of a Gene Co-Expression Network With the Magenta, Brown, and Darkred Modules

According to the relativity of expression among the genes, an express weight index of more than 0.1 was chosen as the edge of the network. The cancer-related module network is shown in **Supplementary Tables 4** and **5**. The co-expression network contains 959 genes in total, which includes 616 genes from the brown module, 65 genes from the darkred module, and 278 genes from the magenta module.

**Figure 3:**
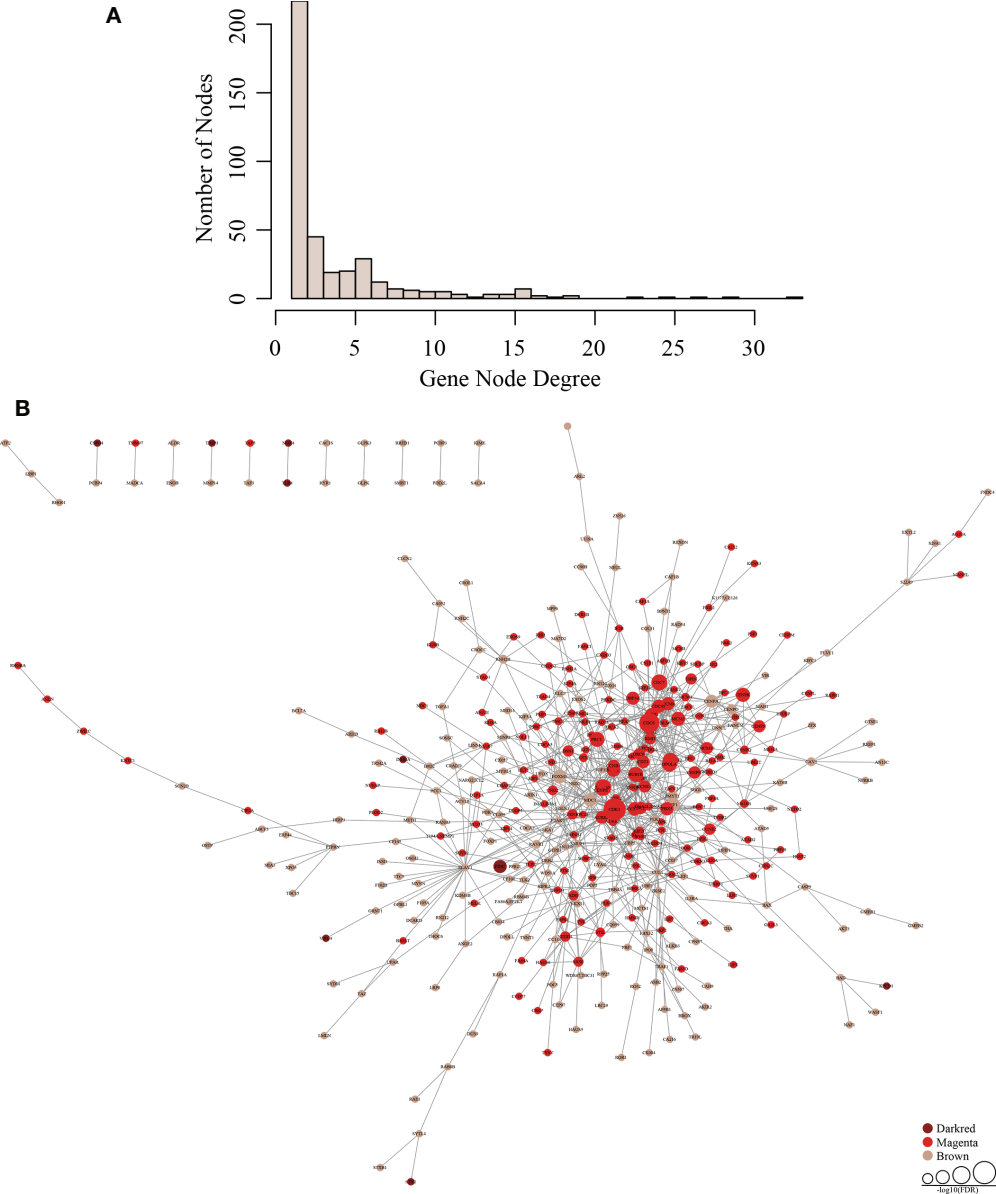
Establishment of a gene co-expression network. **(A)** The distribution of the gene node degrees among the co-expression network of subnet. **(B)** Tumor-specific gene regulation network in three modules (darkred, magenta, and brown).

Next, we downloaded human PPI data from the HIPPIE database to construct a human protein-protein interaction network (**Supplementary Table 6**). The network contains a total of 17,381 nodes, and the average number belonging to neighbor nodes is 19.6. 764 genes are in the network, of which 392 genes are co-expressed and interactive with an average of 1.97 neighbor nodes. The network interactions are listed in **Supplementary Table 7**. The node distribution in the network is shown in **Figure 3A**. The higher the gene node degree is, the fewer the number of nodes there is. From these results, we can conclude that most genes in the network tend to be solitary. Only a few genes have obvious concentrated features, and changed expression of these genes will influence neighboring nodes by co-expressing with them. This illustrates that genes with higher gene node degrees are likely to be important disease-related genes.

Using topological property analysis combined with the PPI network, we calculated the number of co-expressed genes in the co-expression-interactions of each gene subnetwork and constructed a statistical model to conduct functional enrichment analysis. Tumor-specific gene regulation networks in the three modules are shown in **Figure 3B**. There are few genes in the darkred module, the gene node degrees in the brown module are generally low, and the green module shows aggregative features.

### Tumor-Specific Gene Screening, KEGG Enrichment Analysis, and Hierarchical Clustering Analysis

**Figure 4:**
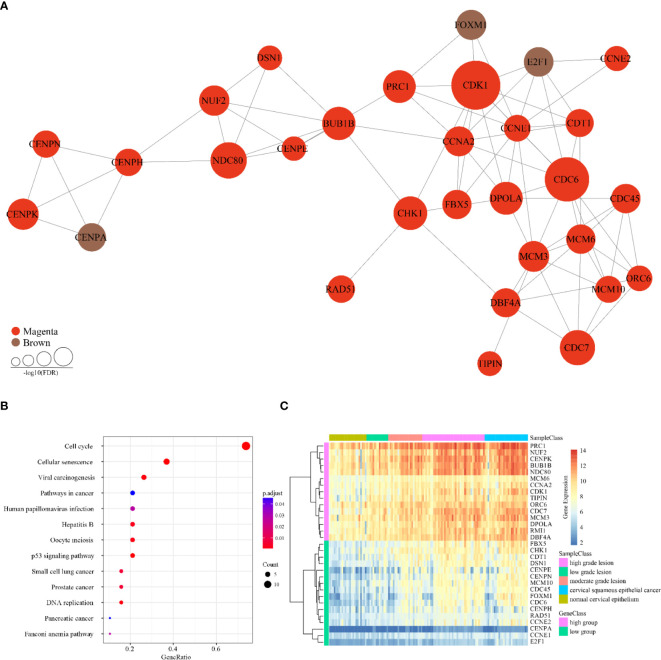
Characteristics of 31 tumor-specific genes. **(A)** Interaction relationships of 31 tumor-specific genes. Three genes belong to the brown module and 28 genes belong to the magenta module. **(B)** KEGG enrichment results of 31 tumor-specific genes. **(C)** Expression clustering analysis of 31 tumor-specific genes. The horizontal axis above represents the samples, using Euclidean distance. The samples were grouped into five sample classes with two gene classes.

In line with the topological properties of genes in the network and the significance of enrichment among the co-expressed genes, 31 genes were determined to be tumor-specific genes by gene profiling. These results are shown in **Table 1**, and their interactions are shown in **Figure 4A**. To determine the functions of these 31 genes, we conducted KEGG analysis. **Figure 4B** shows that these genes are enriched in 13 KEGG pathways, most of which are closely associated with cancer.

**Table 1:** Thirty-one tumor-specific genes by gene profiling.

Gene Symbol	Number of Co-Expression Neighborhood gene	Number of Neighborhood gene	Number of Co-Expression gene	Number of network gene	Co-Expression Neighborhood gene ratio	Fisher’s exact test *p*-value	FDR (False discovery rate)
CCNE1	12	87	392	17,381	0.121212	2.31E-06	0.000839
CDT1	11	60	392	17,381	0.15493	5.08E-07	0.000188
MCM6	15	108	392	17,381	0.121951	1.16E-07	4.39E-05
ORC6	7	26	392	17,381	0.212121	7.24E-06	0.002622
CDK1	33	203	392	17,381	0.139831	4.17E-17	1.63E-14
MCM10	10	55	392	17,381	0.153846	1.80E-06	0.000657
FOXM1	13	72	392	17,381	0.152941	5.45E-08	2.08E-05
DSN1	8	44	392	17,381	0.153846	1.97E-05	0.007069
CENPE	7	33	392	17,381	0.175	2.76E-05	0.00985
BUB1B	16	88	392	17,381	0.153846	1.44E-09	5.56E-07
CENPH	8	28	392	17,381	0.222222	1.08E-06	0.000399
NDC80	16	68	392	17,381	0.190476	5.22E-11	2.03E-08
RMI1	6	17	392	17,381	0.26087	9.24E-06	0.003334
CCNA2	16	119	392	17,381	0.118519	6.61E-08	2.51E-05
POLA1	12	42	392	17,381	0.222222	2.14E-09	8.25E-07
MCM3	19	160	392	17,381	0.106145	2.40E-08	9.18E-06
RAD51	14	112	392	17,381	0.111111	9.75E-07	0.00036
FBXO5	9	29	392	17,381	0.236842	1.26E-07	4.73E-05
CHEK1	17	99	392	17,381	0.146552	9.53E-10	3.70E-07
CENPA	14	102	392	17,381	0.12069	3.51E-07	0.000131
CDC7	11	24	392	17,381	0.314286	1.71E-10	6.67E-08
CENPN	7	19	392	17,381	0.269231	1.28E-06	0.000469
CCNE2	7	29	392	17,381	0.194444	1.34E-05	0.004806
E2F1	16	122	392	17,381	0.115942	9.02E-08	3.42E-05
PRC1	11	33	392	17,381	0.25	2.63E-09	1.01E-06
TIPIN	5	11	392	17,381	0.3125	2.02E-05	0.007246
CDC6	18	50	392	17,381	0.264706	7.03E-15	2.75E-12
NUF2	10	34	392	17,381	0.227273	3.80E-08	1.45E-05
CDC45	10	39	392	17,381	0.204082	1.14E-07	4.31E-05
DBF4	8	21	392	17,381	0.275862	1.77E-07	6.61E-05
CENPK	7	8	392	17,381	0.466667	1.55E-08	5.95E-06

As the **Figure 4C** showed, the cervical cancer samples were grouped into two clusters according to significant differences in their gene expression, referred to as the high and low groups. Next, we subjected the higher abundance group (high group) for KEGG enrichment, for a total of 15 genes (**Table 2**). Most of these genes are associated with the cell cycle, DNA replication, and oocyte maturity, suggesting that these genes are potential tumor biomarkers (**Table 3**).

**Table 2:** Top 15 genes with higher abundance for KEGG enrichment.

ENTREZID	SYMBOL	GENENAME
4175	MCM6	minichromosome maintenance complex component 6
23594	ORC6	origin recognition complex subunit 6
983	CDK1	cyclin dependent kinase 1
701	BUB1B	BUB1 mitotic checkpoint serine/threonine kinase B
10403	NDC80	NDC80, kinetochore complex component
80010	RMI1	RecQ mediated genome instability 1
890	CCNA2	cyclin A2
5422	POLA1	DNA polymerase alpha 1, catalytic subunit
4172	MCM3	minichromosome maintenance complex component 3
8317	CDC7	cell division cycle 7
9055	PRC1	protein regulator of cytokinesis 1
54962	TIPIN	TIMELESS interacting protein
83540	NUF2	NUF2, NDC80 kinetochore complex component
10926	DBF4	DBF4 zinc finger
64105	CENPK	centromere protein K

**Table 3:** Enrichment analysis top genes with higher abundance.

Pathway	GeneRatio	qvalue	geneID
Cell cycle	8/10	2.87e-12	4175/23594/983/701/890/4172/8317/10926
DNA replication	3/10	7.55e-05	4175/5422/4172
Progesterone-mediated oocyte maturation	2/10	0.0296	983/890

### Validation of Prognosis-Related Genes

**Figure 5:**
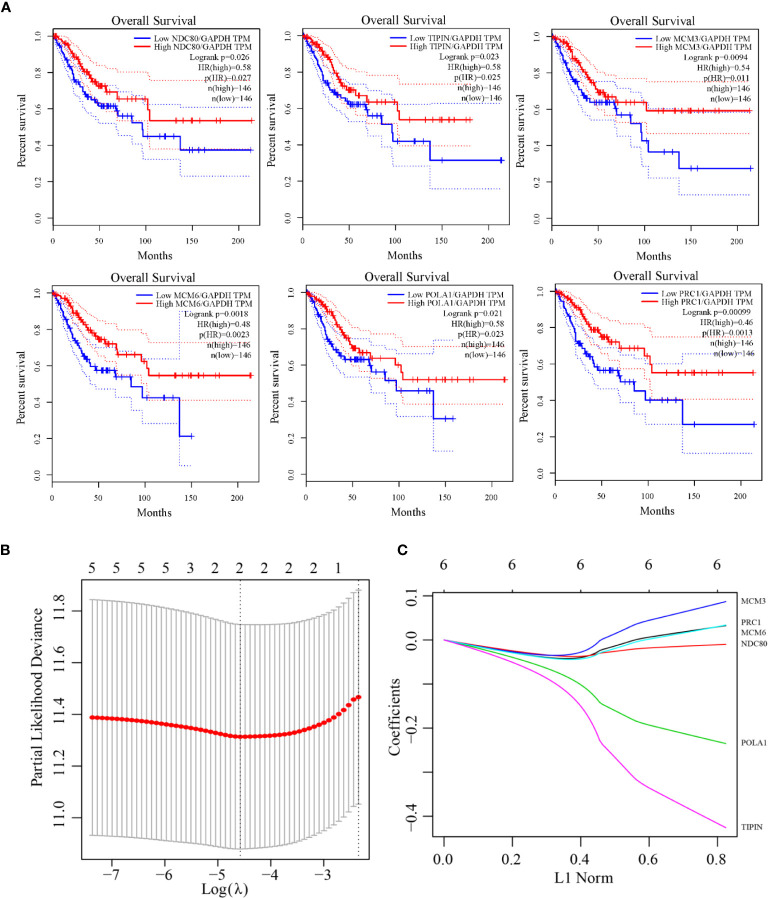
The Kaplan-Meier curves for overall cervical cancer survival in the TCGA RNA-Seq validating dataset and LASSO model construction. **(A)** The prognostic differences of six genes (NDC80, TIPIN, MCM3, MCM6, POLA1, and PRC1) were significant (log-rank test *p-*value = 0.026, 0.023, 0.0094, 0.0018, 0.021, 0.00099). **(B)** Selection of optimal tuning parameter(λ) in the LASSO regression analysis based on 10-fold cross-validation. **(C)** LASSO coefficient profiles of the six prognostic genes.

Univariate Cox regression analysis for each candidate gene was used for relationship of prognosis. Seven genes were filtered in **Table 4** with the threshold value 0.05. Gepia analysis by TCGA RNA-Seq datasets shows in **Figure 5A** that the prognosis of the six genes was significantly different, illustrating that the six genes are of prognostic significance, including NDC80, TIPIN, MCM3, MCM6, POLA1 and PRC1. As indicated by LASSO regression analysis, when λ=0.01028372, the prognostic signature reaches optimal. TIPIN and POLA1 were recognized to be the prognostic genes with the highest accuracy and stability (**Figures 5B, C**).

**Table 4:** Enrichment analysis top genes with higher abundance.

Tag	p.value	HR	Low 95%CI	High 95%CI
MCM6	0.012935	0.699364	0.527528	0.927173
NDC80	0.030895	0.704231	0.512191	0.968273
POLA1	0.007769	0.66108	0.487428	0.896599
MCM3	0.021322	0.725747	0.552418	0.953461
CDC7	0.040749	0.738492	0.552372	0.987323
PRC1	0.020169	0.717347	0.542019	0.949387
TIPIN	0.001188	0.613656	0.45678	0.82441

### Biological Experimental Validation of Genes Expression in Cell Lines

**Figure 6:**
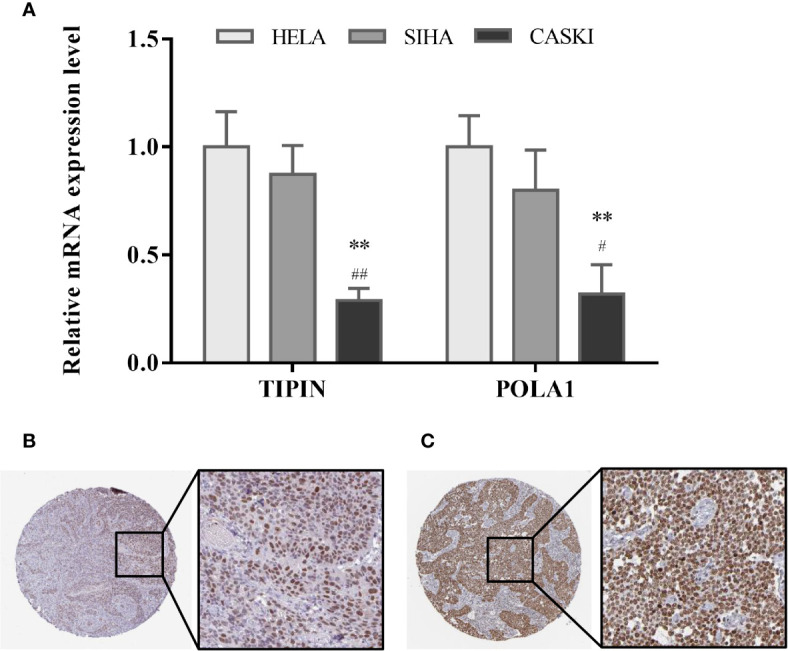
Validation of hub gene expression. **(A)** Relative mRNA expression level of TIPIN and POLA1 in three CC cell lines is conducted by quantitative polymerase chain reaction. Data are presented as the mean ± SD. ^#^P < 0.05, ^##^P < 0.01 vs. SIHA, **P < 0.01 vs. HELA. Expression levels of TIPIN **(B)** and POLA1 **(C)** were confirmed by immunohistochemistry on a translational level using the Human Protein Atlas database.

By quantitative polymerase chain reaction, the relative mRNA expression levels of TIPIN and POLA1 were measured among HELA, SIHA and CASKI cell lines of CC. As **Figure 6A** illustrated, the mRNA expression level of TIPIN and POLA1 both decreased significantly in CASKI cells compared with HELA and SIHA cells.

### Immunohistochemistry Validation of Hub Gene Using the Human Protein Atlas Database

Tissue samples from cervical cancer were picked out in the Human Protein Atlas and the expression levels of TIPIN (**Figure 6B**) and POLA1 (**Figure 6C**) were confirmed by immunohistochemistry. It shows that TIPIN and POLA1 are overexpressed in the CC tissue.

## Discussion

The biological network is a complex network. WGCNA has many distinct advantages over other methods, since the analysis focuses on the association between co-expression modules and clinical traits, and the results have much higher biological significance and reliability (14). The dataset used in this study shows the progression of cervical cancer as a cascade of normal cervical tissue, low grade, moderate grade, high grade, and epithelial cancer (7). Then, the normal samples and four types of cancer samples were formed into a development gradient, and the cancer-related modules were screened out according to the WGCNA.

We integrated the data into a human PPI interaction network, a co-expression interaction network, and analyzed the distribution of gene node degrees to observe the co-expression of genes. Adjacent proteins are often involved in the same disease pathways or biological processes, and the expressions of the core proteins and adjacent proteins were significantly correlated. Therefore, these analyses allow for recognition of biologically-relevant modules and hub genes. They are capable of serving as tumor-specific biomarkers or therapeutic targets. Our approach identified significant co-expression modules meaningfully correlated with the grade and stage of CC and revealed hub genes. The Cancer Genome Atlas (TCGA) is a comprehensive, multi-dimensional database of cancers, which can help to find new therapeutic biomarkers in clinics as oncogenic contributors (15,16). Survival analysis was then used to compare the outcomes associated with gene expression. Six genes, including NDC80, TIPIN, MCM3, MCM6, POLA1, and PRC1, were identified as prognostic indicators for CC.

It has been reported that elevated NDC80 expression may participate in promoting the progression of human hepatocellular carcinoma, and it also leads to poor prognosis in osteosarcoma patients (17,18). TIPIN solves a variety of DNA replication problems (19). Previous studies have found that the expression of TIPIN was higher in the most aggressive and proliferative breast cancer subtypes compared to healthy breast tissue (20). In kidney tumor tissue, the Tim-Tipin complex has downstream consequences over the biochemical properties of the replicative DNA helicase and DNA polymerases (21,22), and it also has implications for colorectal carcinogenesis (23). Minichromosome maintenance (MCM) proteins are an essential regulator of DNA replication (24) and are involved in many cancers. MCM3 upregulates the proliferation of MCF-7 breast cancer cells and H1299 lung cancer cells (25). In another study, MCM3 was found to be a potential marker of cellular proliferation in oral squamous cell carcinoma (26). Moreover, it is also involved in prostate cancer and ovarian cancer (27,28). MCM6 expression has a close correlation with histopathological grades and prognosis in endometrioid endometrial adenocarcinoma and is also implicated in the prognosis of lung cancer (29,30). POLA1 is downregulated and distinctly contributes to the pathways of DNA replication (31). POLA1 is also a candidate target gene for miR-206 in a protein network that regulates carcinogenesis (32). PRC1 has been reported to be involved in cell-fate determination by affecting the gene expression that is involved in genomic instability and malignancy (33,34). Moreover, Shen et al. elucidated a novel PRC1-independent function and revealed a mechanistic rationale for its candidacy as a new prognostic marker and/or therapeutic target in human cancer (35). The above genes participated in the development and progression of related cancers, and our results also indicate that these genes play a vital role in the cascade of cervical cancer progression. At the same time, studies suggest that there is certain relationship between these genes and the biological behavior of CC. NDC80 and MCM6 may be utilized as prospective biomarkers for early detection of cervical cell neoplasia (36). It is consistent with another co-expression analysis in transcriptome sequencing profiles of CC tissues and SiHa cells (37). A research carried by Yukio Ishimi et al. indicated that the synthesis of MCM6 proteins was accelerated in HeLa cells and immunohistochemical studies of surgical materials from human uterine cervix showed that MCM3 is identified to be ubiquitously overexpressed in cancer cells (38). It was reported that SIX1 could function as a master regulator in DNA replication system, including the genes such as MCM6 and POLA1, by both edgeR-Onto and GSEA package. However, TIPIN was identified for the first time in CC in our study. Above all, these genes may become an evaluation index for the grading and prognosis of CC in the future. Future studies will further define the biological mechanisms of these genes during the progression of cervical cancer.

There has been emerging multi-gene biomarkers research in recent years on prognostic signatures of cancer including CC (39,40). Previous literatures have gathered multiple transcriptomic datasets as an integrative multi-omics approach, then the same up/down-regulated transcripts in all datasets were picked up for hub genes mining (41). However, they stayed in the bioinformatics analysis stage and lack experimental validation and accuracy and specificity. An advanced algorithm, using WGCNA to obtain integrative hub genes and an integrated human PPI interaction network-based systemic biology approach, emerged in this study to characterize the gene expression profiles generated from cervical cancer samples. WGCNA is notably useful for the identification of the modules of co-expressed genes that are correlated with clinical traits and consequently biological tumor behavior. Based on the results, the magenta, brown, darkred modules were defined as the most crucial modules for the progression of cervical cancer staging. Six hub genes of potential importance, NDC80, TIPIN, MCM3, MCM6, POLA1 and PRC1, were determined to be significant. They could be used to review, visualize, and analyze tumor genome data in multiple dimensions, and help researchers study the genomics, epidemiology, gene expression, and proteomic events of CC. In order to maintain high accuracy, we applied the glmnet function in R to narrow down the hub-gene range and construct a prognostic signature by LASSO regression analysis. We obtained a relatively refined model with TIPIN and POLA1 as the prognostic genes, which is of highest accuracy and stability. Therefore, it retained subset contraction and better solved the multicollinearity problem in regression analysis.

To identify the two hub-gene signature, qPCR was applied to test the relative mRNA expression level of TIPIN and POLA1 in human CC cell lines, in which SIHA and HELA are the CC primary cell lines, while CASKI is the intestinal-metastatic cell line of CC. TIPIN and POLA1 are likely to be down-regulated in aggressive tumors and linked with better prognosis. It is worth mentioning that the high expression levels of TIPIN and POLA1 were shown to be upregulated by immunohistochemistry in HPA online database. However, the results from the HPA database also have their own limitation. Immunohistochemistry can only show differences in the expression of CC tissue compared to normal cervix tissue, and the conclusion lacks verification. For this reason, we managed to compare CC primary cell line HELA, SIHA with metastatic cell line CASKI for the moment. The results showed that gene expression in metastatic carcinoma was lower than that in cancer primary cells, which indicated that lower gene expression of TIPIN and POLA1 was correlated with poor clinical outcomes. To some extent, the differential expression of the two genes determines the tumor progression mechanisms of the tumor. In our research, we used the WGCNA concept and aimed to construct cancer-related modules based on CC grades, seeking for hub genes of prognostic value. According to a series of analysis, TIPIN and POLA1 were recognized to both participate in cancer progression of CC. Nevertheless, the existing conclusions are still insufficient for the establishment of biological targets, which are recognized in clinical practice universally. To some extent, WGCNA can only integrate big data to illustrate relevant conclusions by providing reference and assisting the progression and prognosis of cervical cancer by combining clinical traits. At present, our study can only support this signature preliminarily by molecular biology experiments. While identification of these two hub genes associated with tumor and metastasis was needed to be further studied for illustration of the relationship between prognosis and biological mechanism, such as CC recurrence, metastasis, and other aspects of tumor biological behavior.

In conclusion, the newly constructed interactive network will likely provide a basis for further investigations of the regulatory mechanisms underlying cervical cancer. This will enable the discovery of promising diagnostic and prognostic biomarkers of cervical cancer, as well as new therapeutic targets.

## Data Availability Statement

Publicly available datasets were analyzed in this study, these can be found in The Cancer Genome Atlas (https://portal.gdc.cancer.gov/), the NCBI Gene Expression Omnibus (GSE63514), and The Human Protein Atlas (HPA) online database.

## Author Contributions

JL, SL, and XY reviewed relevant literature and drafted the manuscript. JL and SL conducted all statistical analyses. All authors contributed to the article and approved the submitted version.

## Funding

This work was funded by the National Natural Science Foundation of China (Grant No. 81773108), Liaoning province Department of Education fund (Grant No. QNZR2020013), as well as the 345 Talent Project of Shengjing Hospital.

## Conflict of Interest

The authors declare that the research was conducted in the absence of any commercial or financial relationships that could be construed as a potential conflict of interest.
